# Acute Chagas Disease Outbreak among Military Personnel, Colombia, 2021

**DOI:** 10.3201/eid2909.230886

**Published:** 2023-09

**Authors:** Hernán Darío Vergara, Carlos H. Gómez, Álvaro A. Faccini-Martínez, Ana Catalina Herrera, María José López, Camila Camacho, Lilian Muñoz, Lissa Cruz-Saavedra, Carolina Hernández, Juan David Ramírez

**Affiliations:** Hospital Militar Central, Bogota, Colombia (H.D. Vergara, C.H. Gómez, Á.A. Faccini-Martínez, A.C. Herrera, M.J. López, L. Muñoz);; Servicios y Asesorías en Infectología, Bogota (Á.A. Faccini-Martínez);; Universidad Militar Nueva Granada, Bogota (C. Camacho);; Universidad del Rosario, Bogota (L. Cruz-Saavedra, C. Hernández, J.D. Ramírez)

**Keywords:** Chagas disease, Trypanosoma cruzi, parasites, vector-borne infections, military personnel, disease outbreaks, Colombia

## Abstract

We report an acute Chagas disease outbreak among soldiers in Colombia. *Trypanosoma cruzi* infection was confirmed through parasitology, serology, and molecular methods. Among 9 affected soldiers, 2 died; 7 were hospitalized and received benznidazole treatment, which produced favorable outcomes. Personnel patrolling rural areas in Colombia could be at increased risk for Chagas disease.

Chagas disease, caused by *Trypanosoma cruzi* parasites, often progresses to a chronic phase that includes cardiovascular, gastrointestinal, and neurologic sequelae (*1*). However, acute forms account for ≈1% of reported cases and can have severe clinical manifestations, especially when orally acquired because of the particularly high parasitic load from this transmission route (*1*). Some populations can be at high risk for infection, including military personnel who are in endemic areas patrolling in rural or jungle environments where the parasite has been documented in multiple reservoirs (*2*,*3*). Although vectorborne transmission is most common, oral transmission has been associated with outbreaks of acute Chagas disease in Latin America and has case fatality rates of 8%–35% (*1*).

In South America, acute Chagas disease outbreaks through oral transmission have been related to food contaminated with triatomine feces or secretions from infected mammals (*1*). Colombia has reported increases in acute Chagas disease due to oral transmission since 1992 (*4*). Up to 35% of acute Chagas disease cases have complications, the most frequent of which are pericardial effusion, myocarditis, and heart failure (*1*,*5*). In rare cases, hemophagocytic lymphohistiocytosis can develop, as reported in the case of a soldier from Colombia (*6*).

We report a case series of acute Chagas disease among military personnel from a base in northeastern Colombia, where the potential risk of enzootic *T. cruzi* transmission was previously reported (*2*). We describe the clinical features observed in the hospital care of infected patients.

## The Study

**Figure 1:**
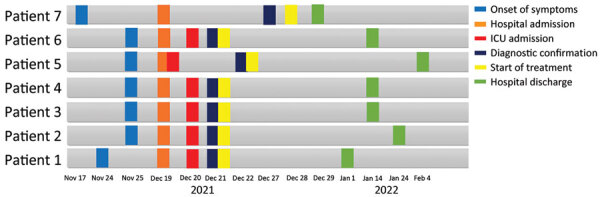
Timeline of acute Chagas disease illness and hospitalizations among military personnel, Colombia, 2021–2022. A group of 9 military personnel had signs and symptoms compatible with acute febrile syndrome, 2 of whom had severe symptoms and died. The remaining 7 patients were admitted to Hospital Militar Central in Bogota, and were treated with benznidazole (5–7 mg/kg/d for 60 days). All 7 had favorable outcomes.

During the third and fourth week of November 2021, a group of 11 military personnel from a base in the municipality of La Jagua de Ibirico, Department of Cesar, Colombia, participated jungle patrols near the base. Within a few days, 9 personnel exhibited signs and symptoms compatible with acute febrile syndrome; 2 persons had severe symptoms and died, and the remaining 7 were transferred to the Hospital Militar Central, a reference military hospital in Bogota, Colombia. Hospitalization dates for the 7 admitted patients ranged from December 19, 2021, through February 4, 2022 (Figure 1).

The 7 patients had no relevant medical history; 6 (86%) required immediate transfer to the intensive care unit for monitoring. All patients had fever; other signs and symptoms included chest pain, dyspnea, abdominal pain, vomiting, and diarrhea (Table). Four (57%) patients required pericardiocentesis for moderate pericardial effusion. None required ventilatory support or vasopressors. We collected clinical and laboratory data through interviews and review of electronic medical records. None of the patients reported seeing triatomines or opossums within the military base facilities where they were located.

**Table:** Clinical features and laboratory test results of acute Chagas disease among military personnel, Colombia, 2021*

Characteristics	Patient 1	Patient 2	Patient 3	Patient 4	Patient 5	Patient 6	Patient 7
No. days fever at hospital admission	26	25	25	25	25	25	33
Symptoms	Fever, asthenia, adynamia, diarrhea, vomiting, chest pain, orthopnea	Fever, asthenia, adynamia, abdominal pain, pleuritic pain, dyspnea	Fever, chills, vomiting, abdominal pain, pleuritic pain, dyspnea	Fever, headache, chills, abdominal pain, diarrhea, vomiting, pleuritic pain, dyspnea	Fever, abdominal pain, diarrhea, vomiting, dry cough, orthopnea	Fever, asthenia, malaise, diarrhea, pleuritic pain, dyspnea	Fever, asthenia, adynamia, diarrhea
ICU admission	Y	Y	Y	Y	Y	Y	N
Pericardial effusion, mL	+, 600	+, 450	+, 760	+, 600	+, 200	+, ND	No effusion
Troponin, pg/mL	1,111	225	722	671	997.3	568	4.7
Echocardiography	LVEF 29%, generalized apical hypokinesia, LVMI 131 g/m^2^	LVEF 54%, hypokinesia of apical predominance, LVMI 112 g/m^2^	LVEF 38%, decreased right ventricular filling pattern without contractility disorder, LVMI 120 g/m^2^	LVEF 28%, generalized hypokinesia, LVMI 150 g/m^2^	LVEF 35%, no contractility disorder, LVMI 120 g/m^2^	LVEF 62%, no contractility disorder, LVMI 104 g/m^2^	LVEF 60%, no contractility disorder, LVMI 72 g/m^2^
EKG	Sinus rhythm, generalized low voltage, and anterolateral repolarization disorder	Sinus rhythm, generalized low voltage, and anterolateral repolarization disorder	Sinus tachycardia, generalized repolarization disorder, and low voltage	Sinus tachycardia, generalized repolarization disorder, and low voltage	Sinus tachycardia, generalized repolarization disorder, and low voltage	Sinus tachycardia, generalized repolarization disorder, and low voltage	Sinus rhythm
Hemoglobin, g/dL	12	10.1	10.6	13	9.5	12.2	15.6
Creatinine, mg/dL	0.94	0.75	0.82	0.92	0.88	0.76	1.04
Thick blood smear†	–	–	–	–	–	–	–
SARS-CoV-2 rapid antigen test‡	–	–	–	–	+	–	–
SARS-CoV-2 qRT-PCR‡	–	–	–	–	+	–	–
ELISA, IgG antibodies to *Trypanosoma cruzi*	+	+	+	+	+	+	–
Strout test	+	–	+	+	NP	NP	–
qPCR for *T. cruzi*, parasites/mL§	+, 0.5	+, 4.55	+, 3.25	+, 5.4	+, 5.8	+, 2.63	+, 0.5
Direct microscopic examination of pericardial fluid¶	+	+	+	+	NP	NP	NP

*EKG, electrocardiography; ICU, intensive care unit; LVEF, left ventricular ejection fraction; LVMI, left ventricular mass index; ND, no data; NP, not performed; qRT-PCR, real-time quantitative reverse transcription PCR; qPCR, quantitative PCR; +, positive; –, negative. †Presence of trypomastigotes. ‡Nasal swab. §Whole blood for *T. cruzi* DNA detection. ¶Detection of trypomastigotes via pericardiocentesis.

**Figure 2:**
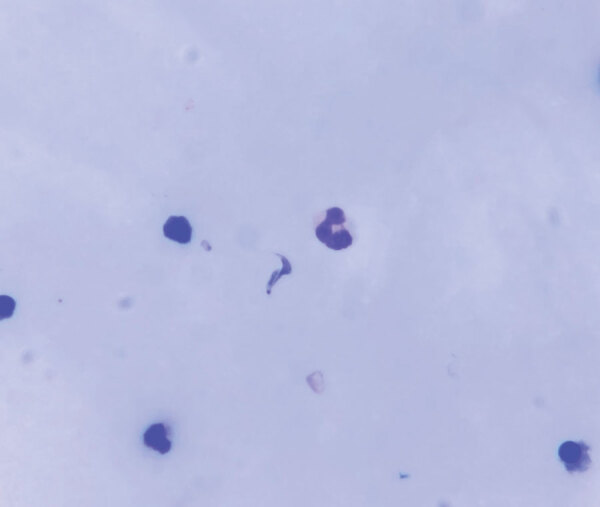
Pericardial fluid smear collected for diagnosis of acute Chagas disease among military personnel, Colombia, 2021. Giemsa-stained pericardial fluid smear of patient 1 shows a *Trypanosoma cruzi* parasite (center). Original magnification ×1,000.

We obtained blood and serum samples from the 7 admitted patients. Overall, diagnosis of acute Chagas disease was made by ELISA serology, Strout concentration method, and molecular tests. In blood samples, we used quantitative PCR to target *T. cruzi* satellite DNA and conventional PCR to target the mini-exon gene. Direct examination of pericardial fluid subsequently revealed parasites (Figure 2). 

After Chagas disease was confirmed, we started all 7 patients on benznidazole treatment (5–7 mg/kg/d for 60 days), and all had favorable outcomes. Informed consent was obtained from the included patients. The study was approved by the ethics committee of the Hospital Militar Central.

## Conclusions

We describe a group of young soldiers without underlying conditions in whom febrile illness progressed toward deterioration in an average of 24 days. Their disease courses correlate with descriptions in the medical literature of the progression of oral acute Chagas disease, which can occur in a range of 3–22 days after infection, depending on the degree of infecting inoculum (*5*,*7*,*8*).

The frequencies of clinical manifestations in the patients in this study are among the highest described in other reports of acute Chagas disease outbreaks (*5*,*7*,*8*). Our patients had fever (100%), abdominal pain (57.1%), diarrhea (71.4%), vomiting (57.1%), chest pain (71.4%), and dyspnea (71.4%) (Table). Pericardial involvement was high (57%) in our patients compared with other reports. In a report on a 2007 outbreak of acute Chagas disease in the Brazilian Amazon, up to 46.2% of the 233 cases had pericardial involvement (*8*). Another report from Colombia in 2021 analyzed 103 cases of acute Chagas disease that occurred in the department of Casanare and found that 34.9% of the patients had >1 complication, which consisted of pericardial effusion, myocarditis, or heart failure (*5*). The high proportion of our patients with cardiac complications might have been the result of a high parasite inoculum, which is more feasible during oral transmission.

Another finding of note is the area of origin of the cases, because a field epidemiologic study of *T. cruzi* circulation was previously conducted in that area and other military facilities in municipalities with historical reports of triatomines and Chagas disease cases (*2*). In that study, a geospatial analysis was conducted to evaluate the coexistence of triatomines and infected mammals in a training base located in La Loma, in the municipality of Jagua de Ibirico (*2*), the same municipality where the cases we report here occurred. However, that study described a low potential risk for *T. cruzi* transmission and the absence of triatomines near the dormitories or kitchens of the military facility (*2*). 

The characteristics of the outbreak we describe, its temporality and the clinical severity of the cases, strongly suggest transmission via the oral route. All affected case-patients were involved in patrol activities in a rural area near the military base, which could have exposed them to a sylvatic genotype of *T. cruzi* that has been reported in Colombia in association with Chagas disease outbreaks caused by oral transmission (*4*). Although none of the patients treated at our institution died, 2 patients from the same outbreak died at the site of origin. That case-fatality rate (22.2%) is consistent with the reported case-fatality rates in acute Chagas disease, which can average 24.4% (*9*). Outbreaks of orally transmitted Chagas disease usually occur during the warmest months of the year, which coincides with the reported dates and estimated temperatures in the geographic area where our patients were during the month of November. Those conditions could favor a higher density of triatomines and a greater number of parasites in triatomine feces, which would increase the probability of food contamination and, therefore, the possibility of oral infection (*10*).

One limitation of this report is the lack of confirmation of the source of the outbreak. In previous studies in Colombia, evidence of *T. cruzi* seropositivity was demonstrated in 1% of the military population studied in 5 departments (*3*), but no similar studies have been conducted in the area where the outbreak cases in this study originated. Despite the reported low vector contact among military personnel, the geographic characteristics of the region where this outbreak originated are similar to areas with higher vector populations, raising the possibility of sylvatic zoonotic oral transmission. 

In summary, our study shows that military personnel could be exposed to *T. cruzi* through oral transmission while patrolling in Chagas disease–endemic areas. Thus, we advise public health and clinical practitioners who care for military personnel to be aware of acute Chagas disease as an additional parasitic zoonotic infection in cases of undifferentiated febrile syndrome associated with cardiac compromise, especially myocarditis or pericardial effusion.
